# Supplementary material for: Conditional chemoconnectomics (cCCTomics) as a strategy for efficient and conditional targeting of chemical transmission
Source: eLife. 2024 Apr 30;12:RP91927. doi: 10.7554/eLife.91927 (PMC11060718; doi:10.7554/eLife.91927)
Supplement: Figure 3—source data 3. [file elife-91927-fig3-data3.zip › Figure 3 source data3 part 2.zip!/Figure 3-Source data 3-part2/1060f2-1-CRISPResso_Report_gl5umwyq.zip!/Nucleotide_percentage_quilt_around_sgRNA_GCCCACTTCCTGGAGTATCG.pdf]

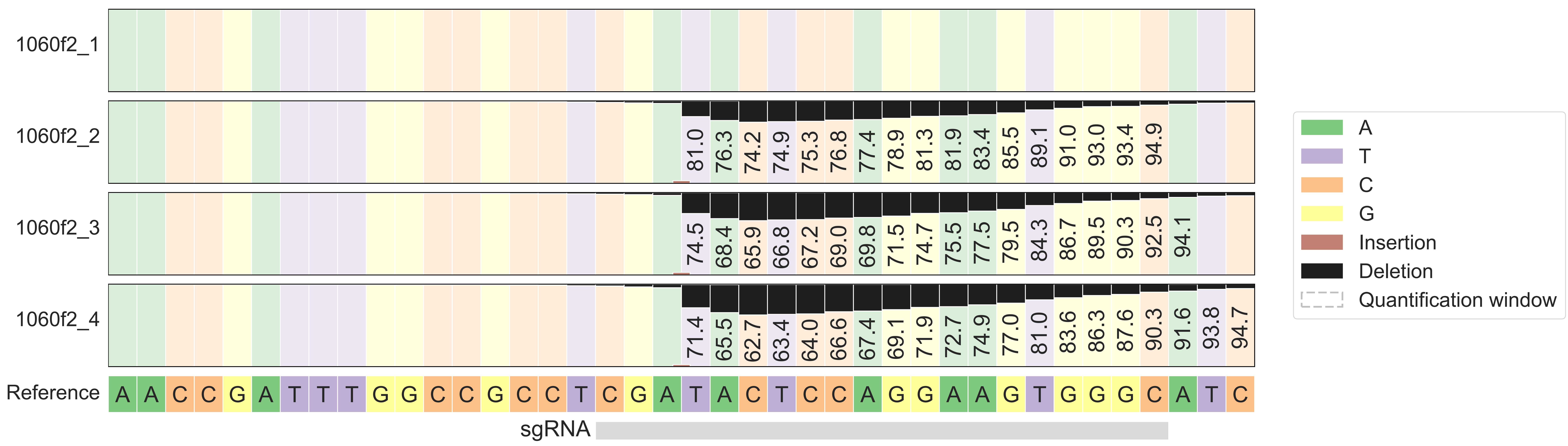
1060f2_1
1060f2_2
81.0 76.3 74.2 74.9 75.3 76.8 77.4 78.9 81.3 81.9 83.4 85.5 89.1 91.0 93.0 93.4 94.9
1060f2_3
74.5 68.4 65.9 66.8 67.2 69.0 69.8 71.5 74.7 75.5 77.5 79.5 84.3 86.7 89.5 90.3 92.5 94.1
1060f2_4
71.4 65.5 62.7 63.4 64.0 66.6 67.4 69.1 71.9 72.7 74.9 77.0 81.0 83.6 86.3 87.6 90.3 91.6 93.8 94.7
Reference
A A C C G A T T T G G C C G C C T C G A T A C T C C A G G A A G T G G G C A T C
sgRNA
A
T
C
G
Insertion
Deletion
Quantification window
